# Molecular watchdogs on genome patrol

**DOI:** 10.7554/eLife.02854

**Published:** 2014-04-29

**Authors:** Gheorghe Chistol, Johannes Walter

**Affiliations:** 1 **Gheorghe Chistol** is in the Department of Biological Chemistry and Molecular Pharmacology, Harvard Medical School, Boston, United Statesgheorghe_chistol@hms.harvard.edu; 2 **Johannes Walter** is an *eLife* reviewing editor, and is in the Howard Hughes Medical Institute and the Department of Biological Chemistry and Molecular Pharmacology, Harvard Medical School, Boston, United Statesjohannes_walter@hms.harvard.edu

**Keywords:** Pif1 family helicases, single molecule analysis, G-quadruplexes, R-loops, DNA helicases, *S. cerevisiae*

## Abstract

By removing various obstacles from single strands of DNA, an enzyme called Pif1 clears the way for other enzymes that act on DNA.

**Related research article** Zhou R, Zhang J, Bochman ML, Zakian VA, Ha T. 2014. Periodic DNA patrolling underlies diverse functions of Pif1 on R-loops and G-rich DNA. *eLife* **3**:e02190. doi: 10.7554/eLife.02190

**Image** A Pif1 DNA helicase reels in single-stranded DNA to unwind a G-quadruplex

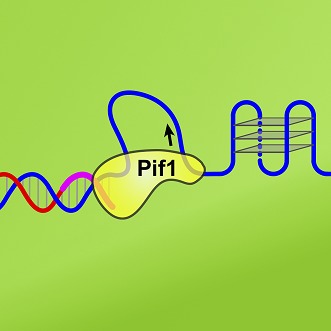

Helicases are enzymes that are best known for unwinding the DNA double helix in preparation for it to be replicated. These helicases, which consist of six protein subunits that form a closed ring, work by sliding along one strand of the DNA molecule. Other helicases function as single subunits. These monomeric helicases, which also work by sliding along DNA or RNA molecules, perform many other functions in cells. To date, there are many aspects of these monomeric helicases that remain poorly understood, including how they specialize to perform different tasks within a cell.

Now, in *eLife*, Taekjip Ha and co-workers at the University of Illinois in Urbana Champaign and Princeton University—including Ruobo Zhou as the first author—have used biophysical techniques to investigate the Pif1 helicase from budding yeast (Zhou et al., 2014). Pif1 is the representative member of a family of monomeric helicases that are conserved from bacteria to humans. Pif1 is ‘a jack of all trades’: it inhibits enzymes that extend the ends of chromosomes (Boule et al., 2005); it helps to link fragments of newly copied DNA (Okazaki fragments) into a continuous strand (Boule and Zakian, 2006; Bochman et al., 2010); and it helps to swap genetic material between chromosomes (Wilson et al., 2013). Pif1 is also thought to prevent the DNA replication machinery from becoming stalled by DNA structures called ‘G-quadruplexes’ (Paeschke et al., 2011, 2013).

To monitor the activity of individual molecules of Pif1, Zhou et al. designed double-stranded DNA molecules with a single-stranded overhang at one end, and used a technique called Förster Resonance Energy Transfer (FRET for short; Roy et al., 2008) to follow how the distance between the two ends of the overhang changed with time (Figure 1). These single-molecule FRET experiments revealed that the Pif1 monomer bound to the junction between the single-stranded and double-stranded DNA, and that it repeatedly ‘reeled in’ the single-stranded overhang, most likely in one-base steps (Figure 1A). Zhou et al. called this activity ‘patrolling’ and showed that an individual Pif1 molecule could complete hundreds of rounds of patrolling (which showed that it was very stably anchored to the junction).

**Figure 1.**
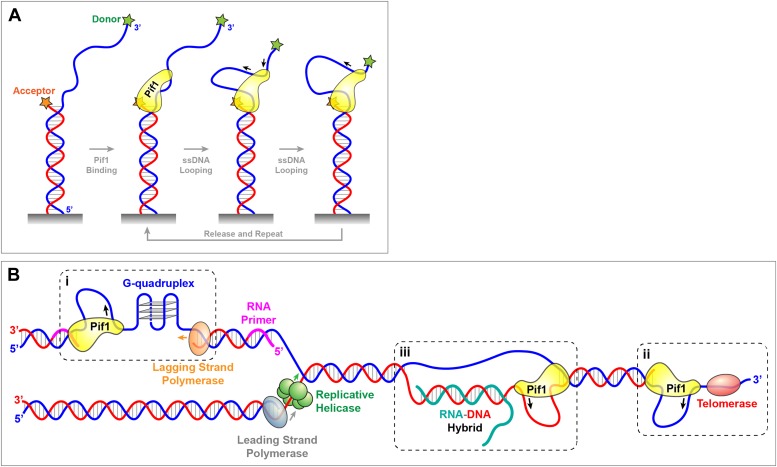
Pif1 patrolling and its diverse genome-maintenance tasks. (**A**) Experimental set-up of the single-molecule experiments in Zhou et al. A helicase substrate consisting of a short DNA double helix (red and blue) with a 3′ overhang (blue) was attached to a glass coverslip (grey). A technique called FRET was used to monitor how the distance between the two ends of the overhang changed over time: this involved adding two organic dyes, a donor (green star) and an acceptor (orange star), to the ends of the overhang and recording how the amount of light emitted by the donor and the acceptor changed with time. Zhou et al. found that Pif1 anchored itself to the junction between the double-stranded DNA and the overhang, and periodically patrolled the single-stranded DNA (ssDNA) overhang by repeatedly reeling it in and forming loops. (**B**) The patrolling activity discovered by Zhou et al. provides a common basis for the diverse functions performed by Pif1 in living cells. (i) It unwinds G-quadruplexes in G-rich regions and facilitates the joining of the Okazaki fragments synthesized by the lagging strand polymerase. (ii) It inhibits the activity of telomerases at double-stranded DNA breaks and also at the ends of chromosomes. (iii) Pif1 also unwinds hybrids of RNA (shown in dark green) and DNA at so-called R-loops.

How does this patrolling activity relate to the multitude of tasks that Pif1 performs in a cell? Zhou et al. challenged the helicase with three obstacles that it might encounter in living cells: double-stranded DNA, RNA-DNA hybrids, and G-quadruplexes. This last obstacle—which forms when a stretch of DNA containing several consecutive guanine or ‘G’ bases folds back upon itself to form a stable three-dimensional structure—can prevent gene expression and slow down DNA replication. Zhou et al. reveal that Pif1 can efficiently unfold any G-quadruplexes that it encounters as it patrols single-stranded DNA. Although these structures rapidly refold after the Pif1 has passed, repeated patrolling by Pif1 ensures that G-quadruplexes remain unfolded.

Pif1 is known to facilitate the replication of DNA sequences that are rich in G bases and therefore prone to forming G-quadruplexes (Paeschke et al., 2011, 2013). Pif1 might do this by anchoring itself to an end of a newly replicated DNA fragment and clearing out G-quadruplexes that would otherwise obstruct the DNA replication machinery (Figure 1B). Similarly, Pif1 could periodically patrol single-stranded DNA at the ends of chromosomes to unwind G-quadruplexes and evict the enzymes that extend these regions (Boule and Zakian, 2007; Paeschke et al., 2013).

Zhou et al. also found that monomeric Pif1 can slowly unwind a RNA-DNA hybrid, but cannot unwind double-stranded DNA. Given that RNA-DNA hybrids are at least as stable as a DNA double helix (Lesnik and Freier, 1995), this finding supports previous work which suggested that Pif1 specifically recognizes and unwinds RNA-DNA hybrids (Figure 1B; Boule and Zakian, 2007). Zhou et al. also found that increasing the concentration of the enzyme could enable Pif1 to unwind double-stranded DNA, but suggest that this was due to multiple copies of Pif1 working together—something that has been observed for other monomeric helicases (Lohman et al., 2008).

Eukaryotic genomes encode a large number of monomeric helicases (Lohman et al., 2008), which suggests that these enzymes each perform specialized tasks. To test this idea, Zhou et al. compared Pif1 with another monomeric helicase called PcrA, which also translocates along single-stranded DNA and displaces proteins bound to this DNA (Park et al., 2010). Although PcrA also patrolled DNA, it could not disrupt G-quadruplexes—indicating that periodic patrolling of single-stranded DNA alone is not sufficient to unwind G-quadruplexes. The findings of Zhou et al. also suggest that monomeric helicases possess unique adaptations suited for their own specialized task.

Zhou, Ha and colleagues have uncovered a basic mechanism by which helicases belonging to the Pif1 family might carry out a wide range of genome-maintenance tasks. In light of these findings, several questions arise: Do individual molecules of Pif1 work in the same way in living cells? Can multiple copies of Pif1 join forces and work together in vivo and how is this process regulated? It will be interesting to know if Pif1 can patrol far enough to span the distance between neighboring Okazaki fragments. Moreover, can the helicase patrol when anchored to an RNA-DNA hybrid, as found at the 5′ end of Okazaki fragments?
